# Pretreatment of sweet sorghum straw and its enzymatic digestion: insight into the structural changes and visualization of hydrolysis process

**DOI:** 10.1186/s13068-019-1613-6

**Published:** 2019-11-23

**Authors:** Miaoyin Dong, Shuyang Wang, Fuqiang Xu, Junkai Wang, Ning Yang, Qiaoqiao Li, Jihong Chen, Wenjian Li

**Affiliations:** 1 Institute of Modern Physics, Chinese Academy of Sciences, 509 Nanchang Rd., Lanzhou, 730000 Gansu People’s Republic of China; 2 Institute of Biology, Gansu Academy of Sciences, 197 Dingxi South Rd., Lanzhou, 730000 Gansu People’s Republic of China; 3 College of Life Sciences, University of Chinese Academy of Sciences, No. 19A Yuquan Road, Beijing, 100049 People’s Republic of China; 4 College of Physics and Electronic Engineering, Northwest Normal University, Anning Rd., Lanzhou, 730000 Gansu People’s Republic of China; 5 College of Life Sciences, Northwest Normal University, Anning Rd., Lanzhou, 730000 Gansu People’s Republic of China

**Keywords:** Sweet sorghum straw, Pretreatment, Structural characterization, Enzymatic hydrolysis process, Visualization

## Abstract

**Background:**

The efficient utilization of lignocellulosic biomass for biofuel production has received increasing attention. Previous studies have investigated the pretreatment process of biomass, but the detailed enzymatic hydrolysis process of pretreated biomass remains largely unclear. Thus, this study investigated the pretreatment efficiency of dilute alkali, acid, hydrogen peroxide and its ultimate effects on enzymatic hydrolysis. Furthermore, to better understand the enzymatic digestion process of alkali-pretreated sweet sorghum straw (SSS), multimodal microscopy techniques were used to visualize the enzymatic hydrolysis process.

**Result:**

After pretreatment with alkali, an enzymatic hydrolysis efficiency of 86.44% was obtained, which increased by 99.54% compared to the untreated straw (43.23%). The FTIR, XRD and SEM characterization revealed a sequence of microstructural changes occurring in plant cell walls after pretreatment, including the destruction of lignin–polysaccharide interactions, the increase of porosity and crystallinity, and reduction of recalcitrance. During the course of hydrolysis, the cellulase dissolved the cell walls in the same manner and the digestion firstly occurred from the middle of cell walls and then toward the cell wall corners. The CLSM coupled with fluorescent labeling demonstrated that the sclerenchyma cells and vascular bundles in natural SSS were highly lignified, which caused the nonproductive bindings of cellulase on lignin. However, the efficient delignification significantly increased the accessibility and digestibility of cellulase to biomass, thereby improving the saccharification efficiency.

**Conclusion:**

This work will be helpful in investigating the biomass pretreatment and its structural characterization. In addition, the visualization results of the enzymatic hydrolysis process of pretreated lignocellulose could be used for guidance to explore the lignocellulosic biomass processing and large-scale biofuel production.

## Background

The ever-increasing demand for energy and environmental concern is forcing the exploration of sustainable energy from the renewable substrates, aiming at reducing our dependence on fossil fuels [1, 2]. Lignocellulose is one of the most abundant organic materials on Earth, including energy crops, agricultural and forestry residues, and its conversion to a variety of valuable products such as liquid fuels and other carbon-based materials has attracted considerable concern [3, 4]. Sweet sorghum (*Sorghum bicolor*), a energy corp in China that can be cultivated in harsh growth conditions, is a promising candidate for generating abundant plant biomass [5].

The native biomass has a complex hierarchical and heterogeneous structure, which is composed of cellulose, hemicellulose, lignin, and other polysaccharides [6]. Unfortunately, the natural recalcitrance of lignocelluloses, particularly the non-polysaccharide aromatic polymer lignin, posed significant resistance to enzymatic and microbial deconstruction in biomass processing [7]. Therefore, the exploration of efficient and selective pretreatment systems and hydrolysis processes was necessary to achieve the efficient utilization of lignocellulose substrates. To improve the saccharification efficiency of natural lignocelluloses, different pretreatment technologies including chemical, physical and biological approaches have been initiated prior to enzymatic hydrolysis [8]. The main goals of pretreatment were to reduce biomass recalcitrance via minimizing the impact of lignin–hemicellulose matrix and producing digestible cellulose residues. In our previous study, the effect of heavy-ion beam irradiation pretreatment on enzymatic hydrolysis of sweet sorghum straw was investigated. We found that the heavy-ion beam irradiation caused the transformation of polymorphs (*I*_α_ → *I*_β_) of cellulose *I*, thereby increasing the enzymatic digestibility of the biomass [9]. However, this irradiation pretreatment requires a sophisticated accelerator device, which also means high cost and limits its industrial application. In addition, biological pretreatment is a relatively time-consuming process, and its hydrolysis rate was lower as compared to other technologies [10, 11]. However, chemical pretreatment, such as using dilute alkali, acid and oxidant, is an economical and efficient pretreatment process that uses relatively low temperatures and pressures [10, 12].

Although many of the pretreatment processes of biomass have been widely studied, the detailed enzymatic hydrolysis process of pretreated biomass remains largely unclear. The visualization techniques and cellulase protein labeling approaches were implemented to represent the detailed information of accessibility and digestibility of cellulase to pretreated biomass [13, 14]. He et al. [15] investigated in situ visualization in lignocellulosic hydrolysis via making a fusion enzyme with green fluorescence protein (GFP). They found that the absorption ability of enzyme protein could not consistently reflect the digestibility of lignocellulose. The possible reason is that the binding and hydrolysis efficiency of the fusion enzyme might be affected by the inserted GFP in that the size of GFP is not negligible [16]. Nevertheless, a deeper understanding of the enzymatic hydrolysis process of pretreated biomass is necessary to enhance the lignocellulosic biomass processing [17].

In this study, we investigated the various pretreatment efficiencies (NaOH, H_2_SO_4_, H_2_O_2_) and its ultimate effects on enzymatic hydrolysis. Then, FTIR, XRD and SEM were used to characterize the effects of pretreatment and enzymatic hydrolysis on the microstructure of the biomass. Furthermore, to better understand the enzymatic hydrolysis process of alkali-pretreated biomass, the real-time imaging analysis of the hydrolysis process was carried out. In addition, the CLSM coupled with fluorescent-labeling techniques was used to visualize the spatial changes in the accessibility and digestibility of cellulase to the pretreated biomass during hydrolysis.

## Materials and methods

### Sweet sorghum straw

The sweet sorghum straw was collected from the experimental field of the Institute of Modern Physics, Gansu (latitude 37.93° N, longitude 102.63° E), China. After drying at 60 °C in an oven for 48 h, the straw was ground in a milling machine and passed through size 40 mesh sieves. To visualize the enzymatic hydrolysis process, the transverse slices (20 µm and 10 µm in thickness) of sweet sorghum stem (fifth internode) were prepared using Leica microtome (RM2265, China). Then, the transverse sections were dewaxed in xylene and rehydrated with graded ethanol as described in Kim et al. [18] and dried at 60 °C to a constant weight.

### Preparation of cellulase enzyme

The cellulase enzymes (*T. longibrachiatum* LC-M4) were provided by the Institute of Modern Physics and its filter paper activity was 66.7 FPU/g. For the cellulase labeling, 1 mg of fluorescein isothiocyanate (FITC) was dissolved in 1 mL anhydrous dimethyl sulfoxide (DMSO), then the cellulase (20 mg) was added to the FITC solution and the labeling reaction was performed in the dark at 4 °C for 8 h. After incubation, the free dyes were removed by the centrifuging process using 10 kDa centrifugal membrane concentrator (Millipore, China).

### Pretreatment of sweet sorghum straw

H_2_SO_4_ at a concentration of 2% (v/v) was used to pretreat 5 g ground SSS samples at a solid loading of 10% (w/v) in 250 mL Erlenmeyer flasks. Pretreatment was performed in an autoclave at 121 °C for 1 h. 2% NaOH (w/v) and 10% hydrogen peroxide (v/v) pretreatment was carried out at 10% (w/v) biomass loading in 250 mL Erlenmeyer flasks and at 100 °C for 1 h, respectively. The transverse section slices were pretreated with 2% NaOH (w/v) in a glass Petri dish (90 mm) at the same conditions as described above. Then the pretreated samples were washed with distilled water until the pH was neutral and dried at 60 °C to a constant weight.

### Enzymatic hydrolysis of sweet sorghum straw

The enzymatic hydrolysis was performed as described in our previous study [19]. Briefly, the SSS with or without pretreatment, cellulase (9 FPU/g substrate) and citric acid buffer solution (50 mM, pH 4.8) were added to 150 mL Erlenmeyer flasks at a substrate mass concentration of 1 g/20 mL. Then, the hydrolysis reaction was carried out with a stirring rate of 150 rpm at 50 °C for 72 h. After enzymatic hydrolysis, 1 mL of the sample was centrifuged at 8000 rpm for 10 min and the total glucose contents were measured by the HPLC method [20]. The enzymatic hydrolysis efficiency was calculated as described in our previous study [19]. Based on the delignification capability and hydrolysis efficiency for SSS, the best pretreatment method was used for further enzymatic hydrolysis process study.

### Analytical methods

The contents of cellulose, hemicellulose and lignin in the samples before and after pretreatment were calculated by following the National Renewable Energy Laboratory (NREL) method as described in Sluiter et al. [21]. The surface morphology of pretreated and enzyme-hydrolyzed samples was imaged using NanoSEM 490 SEM (FEI.Co, USA) with an accelerating voltage of 15 kV at magnifications of 1000 [22]. The crystalline phases of pretreated and hydrolyzed straws were characterized by X-ray diffractogram (XRD, Bruker D5005, Karlsruhe, Germany). The samples were scanned from 10° to 40° with a step size of 0.05° and the crystallinity index (CrI) was determined by following “Segal” method (Eq. 1) as described in Segal et al. [23].

1 $${\text{CrI}}\left( \% \right) = {{\left( {I_{002} - I_{\text{am}} } \right)} \mathord{\left/ {\vphantom {{\left( {I_{002} - I_{\text{am}} } \right)} {I_{002} \times 100\% }}} \right. \kern-0pt} {I_{002} \times 100\% }},$$

where *I*_002_ is the highest peak intensity at 2*θ* = 22° and *I*_am_ is the intensity of amorphous portion at 2*θ *= 18°.

In addition, the FTIR spectrometer (Bruker Tensor 27, Germany) was used for revealing the changes of the functional groups in pretreated and hydrolyzed straws. Samples were prepared by grinding with KBr at a ratio of 1:100 (w/w) and pressing into pellets. The spectra were recorded within a range of 400–4000 cm^−1^ with a resolution of 4 cm^−1^ and 32 scans per sample [24].

### Real-time imaging analysis on the enzymatic hydrolysis process of alkali-pretreated SSS

The enzymatic hydrolysis of alkali-pretreated transverse sections was carried out in a glass Petri dish (35 mm) at room temperature. Enzyme loading was estimated roughly as 9 FPU/g substrate based on the average weight of stem transverse sections. For real-time imaging of the enzymatic hydrolysis process, the bright-field light microscopy (Olympus BX53, Japan) was used and individual images were taken with time intervals of 60 min in the same area. In addition, the digestion movies were recorded at minute time intervals in the same area.

### Visualization of the spatial accessibility and digestibility of cellulase to alkali-pretreated SSS

For cell wall binding, 100 µL labeled enzymes were added to the pretreated stem transverse section and incubated on slides at room temperature and rinsed three times with PBS buffer (pH 7.4, 10 min per time). Then the confocal laser scanning microscopy (CLSM, LSM 700, ZEISS, Germany) was used for observing the accessibility and digestibility of labeled cellulase on the surface of stem transverse sections. The labeled cellulase was excited by 488 nm laser and detected by a 515/30 nm emission filter, and the autofluorescence of straw was excited by 543 nm laser and detected by 605/75 nm emission filter [14]. Multi-track image acquisition techniques were used to minimize cross talk of fluorescence emission. All CLSM images were recorded at the same conditions and analyzed using Image J (http://rsb.info.nih.gov/ij/) by the following procedure: “Open” the original CLSM images with Image J → “Analyze” → “Measure”, and then record the intensities of dye-labeled fluorescence and autofluorescence, respectively. The relative intensities of dye-labeled fluorescence and autofluorescence were expressed as percentages compared with the intensities of fluorescence in untreated samples that were designated as 100%, respectively. For quantitative analysis of cellulase binding, the average fluorescence intensities were calculated based on a total of ten images from at least five independently labeled tissue sections.

### Statistical analysis

The statistical analyses were performed using the Origin 9.0 and SPSS 20.0 (SPSS V20.0). All presented values are the means of at least three replicates. Tukey’s test was employed for different sample means and significance was declared at *P* < 0.05.

## Results and discussion

### Analysis of chemical compositions and enzymatic hydrolysis efficiency

The contents of cellulose, hemicellulose and lignin in biomass are crucial factor for bioconversion. Generally, these compositions vary in amounts because of the difference in growth conditions and species [25]. In this study, the untreated SSS contained 37.74% cellulose, 28.07% hemicelluloses and 21.48% lignin (Table 1). The hemicellulose and lignin contents of SSS were much higher than other agro-residues, such as rice straw and wheat straw [26, 27]. These results suggested that an appropriate pretreatment was necessary to produce fermentable sugars using the SSS as feedstock.

**Table 1:** Chemical composition and enzymatic hydrolysis efficiency of sweet sorghum straw before and after pretreatment

Samples	Chemical composition (%)^a^			Enzymatic hydrolysis efficiency (%)
	Cellulose	Hemicellulose	Lignin	
Untreated [19]	37.74 ± 1.61	28.07 ± 1.12	21.48 ± 0.63	43.23 ± 0.9
2% NaOH [19]	71.36 ± 3.2	16.15 ± 0.84	6.29 ± 0.07	86.44 ± 3.42
2% H_2_SO_4_	57.80 ± 0.95	11.76 ± 0.27	17.83 ± 0.8	75.07 ± 1.08
10% H_2_O_2_	54.60 ± 2.46	24.53 ± 0.3	11.57 ± 0.19	66.60 ± 1.37

^a^Based on dry weight of samples

The contents of cellulose were significantly increased after pretreatment and the highest contents of 71.36% were obtained in 2% NaOH-pretreated SSS (Table 1). This increase of cellulose contents indicated that a large number of cellulose located in cell walls were released from the hemicellulose–lignin matrix after pretreatment [27]. The lignin contents in 2% NaOH-treated SSS were significantly decreased to 6.29%, which were suitable for further enzymatic hydrolysis. 2% H_2_SO_4_ pretreatment resulted in efficient removal of hemicellulose components in SSS, while 10% H_2_O_2_ is the least effective in delignification. According to Moxley et al. [28], the hemicellulose is generally diffused into the interfibrillar space through the fiber pores and cross-linked the cellulose microfibrils and lignin, which reduced the accessibility of the cellulose to cellulases. They demonstrated that the dilute acid pretreatment significantly improved the enzyme accessibility through xylan solubilization and lignin degradation.

As can be seen from Table 1, the enzymatic hydrolysis efficiency was significantly improved after pretreatment and 2% NaOH-pretreated sample exhibited the highest efficiency (86.44%), followed by 2% H_2_SO_4_ (75.07%) and then 10% H_2_O_2_ (66.6%) pretreatment. Ioelovich et al. [29], who applied both NaOH and dilute H_2_SO_4_ to four materials (poplar, switchgrass, corncobs and rice straw), reported alkali pretreatment to be more efficient in terms of delignification, sugar yields, and biomass utilization rate. In this study, the increase of hydrolysis efficiency in H_2_O_2_ pretreatment might be due to the oxidative delignification that can increase lignin solubilization and cellulose availability [30, 31]. It was noted that the hydrolysis efficiency of SSS was negatively related to the contents of lignin or hemicellulose, which affects the enzymatic hydrolysis by binding to cellulose, thereby decreasing the possibility of cellulose contacting cellulase [32].

### Effect of pretreatment and enzymatic hydrolysis on the structures of SSS

#### Fourier transform infrared (FTIR) spectroscopy analysis

**Fig. 1:**
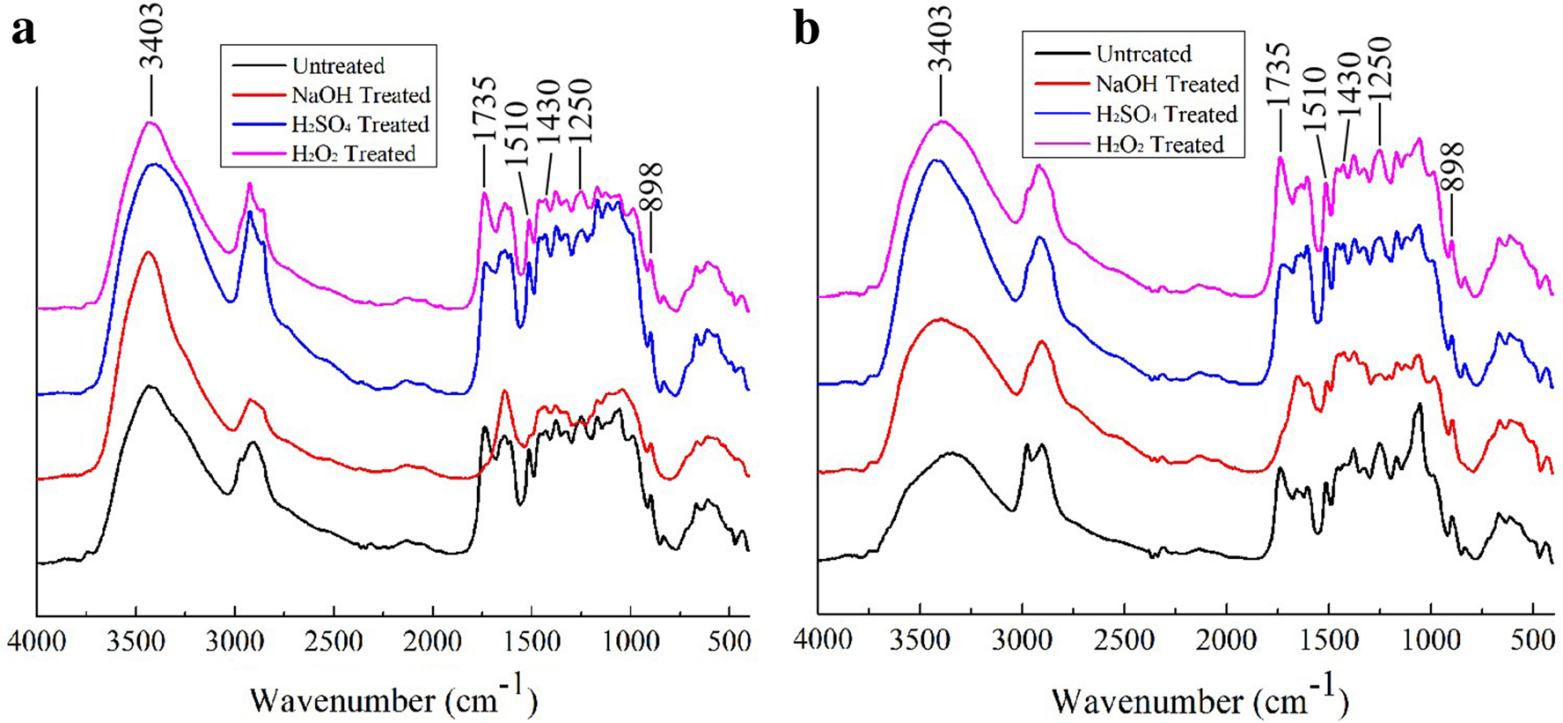
FTIR spectroscopy of SSS after pretreatment (**a**) and enzymatic hydrolysis (**b**)

Figure 1 shows the changes in the chemical structure of SSS after pretreatment and enzymatic hydrolysis. The spectral band at 3403 cm^−1^ was attributed to the hydroxyl group (–OH) stretching vibrations in hemicellulose and hydrogen-bonded water molecules absorbed by the polymers [33, 34]. Thus, the broad band at 3403 cm^−1^ could not clearly resolve the vital information about the changes in biomass structure during pretreatment and enzymatic hydrolysis due to the overlap of –OH stretching vibrations from hemicellulose and hydrogen-bonded water [35]. The band at 1735 cm^−1^ represents the characteristic peaks of the hemicellulose–lignin complex. The intensity of 2% H_2_SO_4_-pretreated SSS was decreased, while it disappeared in 2% NaOH-pretreated SSS, indicating that the alkaline pretreatment could not only hydrolyze the lignin fraction but also extract some hemicellulose [26]. The peaks at 1510 cm^−1^ (guaiacyl ring from lignin) almost disappeared in NaOH-pretreated SSS, which indicated the occurrence of extensive delignification. McMillan et al. [36] demonstrated that the saponification reactions between NaOH and intermolecular ester bonds in lignin were the key factors causing lignocellulosic structure changes after NaOH pretreatment, which contributes to lignin removal and cellulose release, while this spectral band at 1510 cm^−1^ could be seen in untreated, 2% H_2_SO_4_ and 10% H_2_O_2_-treated SSB, which were there even after enzymatic hydrolysis. These results indicated that the lignin components of SSS cannot be removed by the acid or oxidant pretreatment, and cannot also be digested by cellulase.

The hemicellulose peaks (acetyl groups C–O) were obviously identified at 1250 cm^−1^ in untreated and 10% H_2_O_2_-treated SSB, and an increase in intensity of untreated, 2% H_2_SO_4_- and 10% H_2_O_2_-treated SSB was observed after hydrolysis indicated that the acetyl groups in hemicellulose were partially exposed due to cellulose digestion. A previous study reported that the hemicellulose backbone and its side chains in cell walls could inhibit the formation of productive binding between the catalytic domain of cellulases and cellulose, thereby limiting the cellulose accessibility [37]. In addition, the acetyl groups can increase the diameter of cellulose chains, thus slowing the enzymatic hydrolysis rate by increasing the steric hindrance of enzymes [38]. Therefore, the hemicellulose was typically thought to impede the enzymatic digestion of cellulose via forming a physical barrier on the cellulose microfibril surface [39, 40], but the barrier of hemicellulose seems to be less important compared to the effect of lignin in that the hemicellulose is easy to remove during enzymatic hydrolysis (hemicellulase) or the pretreatment process [41]. The bands at 1430 cm^−1^ and 898 cm^−1^ were labeled as cellulose I and the mixture of cellulose II and amorphous cellulose absorption band, respectively [42, 43]. It was reported that the absorption band at 898 cm^−1^ was attributed to the C–O–C stretching vibrations of β-(1-4)-glycosidic linkage in cellulose II and amorphous cellulose [44]. Thus, the band at 898 cm^−1^ was resolved to be that of the mixture of cellulose II and amorphous cellulose. The decrease in intensity at 898 cm^−1^ and increase in intensity at 1430 cm^−1^ after pretreatment were mainly caused by the partial removal of lignin and hemicellulose from the biomass matrix, thereby releasing cellulose [44]. A further decrease in intensity at 898 cm^−1^ could be seen after hydrolysis in all pretreated SSS, because the more amorphous cellulose was hydrolyzed by cellulase compared with the untreated SSS.

#### X-ray diffraction (XRD) analysis

**Fig. 2:**
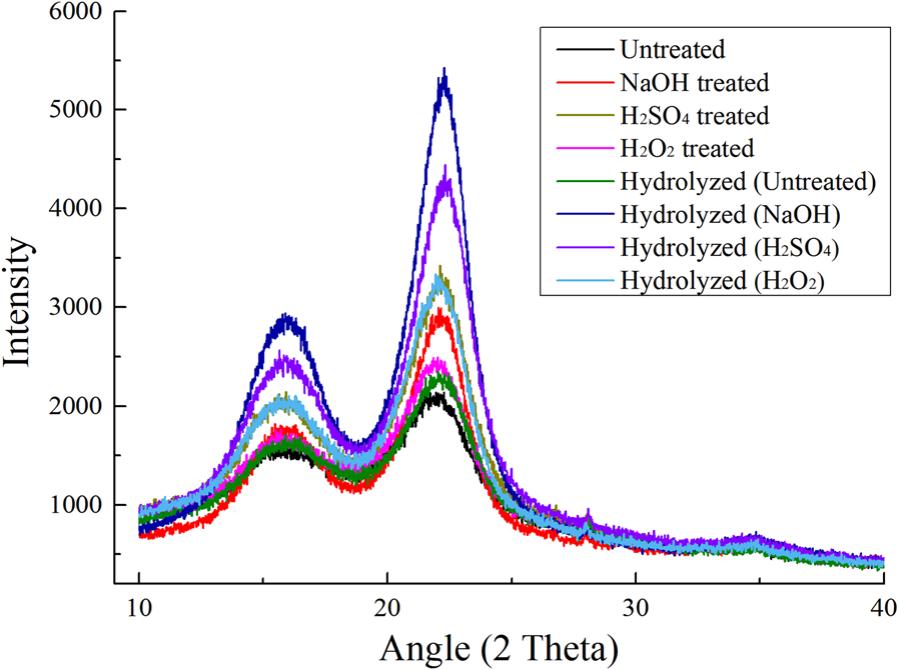
XRD images of SSS after pretreatment and enzymatic hydrolysis

The crystalline feature, as the structural property of lignocellulose, plays an important role in the enzymatic hydrolysis and bioconversion of the biomass [24]. As shown in Fig. 2, the peaks at 16.3° and 22.0° were significantly higher in all pretreated and hydrolyzed SSS (especially in alkali-pretreated and hydrolyzed SSS) than in the untreated sample. The crystallinity index (CrI) was employed to elaborate the crystalline degree of lignocellulose. The CrI of untreated and 2% NaOH-, 2% H_2_SO_4_- and 10% H_2_O_2_-treated SSS were 45.50%, 69.30%, 66.45% and 53.06%, respectively (Table 2). The increase of CrI in pretreated SSS was attributed to the removal of hemicellulose and lignin, both of which are amorphous in the native biomass [45]. A previous study reported that the chemical pretreatment could increase the crystallinity of the lignocellulosic biomass due to the removal of hemicellulose or lignin components [45]. Kainthola et al. [46] reported that the fungal pretreatment of rice straw decrystallized the crystalline part of the substrate, thereby decreasing the crystallinity. These differences may be related to the types of biomass, the conditions of pretreatment and actions of cellulase.

**Table 2:** Crystallinity indices of SSS undergoing pretreatment and enzymatic hydrolysis

Samples	Crystallinity index (CrI)	
	Pretreatment	Enzymatic hydrolysis
Untreated	45.50	53.90
2% NaOH	69.30	77.47
2% H_2_SO_4_	66.45	71.68
10% H_2_O_2_	53.06	64.95

Interestingly, the CrI of untreated and pretreated SSS were further increased after enzymatic hydrolysis when compared with the raw and only pretreated SSS (Fig. 2). We observed that the CrI of 2% NaOH-, 2% H_2_SO_4_- and 10% H_2_O_2_-pretreated SSS increased up to 77.47%, 71.68% and 64.95% from 53.90% of untreated SSS after enzymatic hydrolysis, respectively (Table 2). Because the CrI measures the relative fraction of crystalline cellulose in the total solid, it is widely impacted by the presence of lignin and hemicellulose, crystalline and amorphous regions in cellulose [37]. Zhang et al. [47] reported that a slower hydrolysis of crystalline cellulose as compared to amorphous cellulose would increase the percentage crystallinity of the hydrolyzed biomass. Therefore, the further increase of CrI in hydrolyzed SSS was attributed to the digestion of amorphous cellulose released from the lignin and hemicellulose matrix after pretreatment. After hydrolysis, the highest CrI in alkali-pretreated SSS (77.47%) indicated the high delignification capability of alkali pretreatment, thereby improving the enzymatic hydrolysis efficiency (Table 1).

#### Scanning electron microscopy (SEM) analysis

**Fig. 3:**
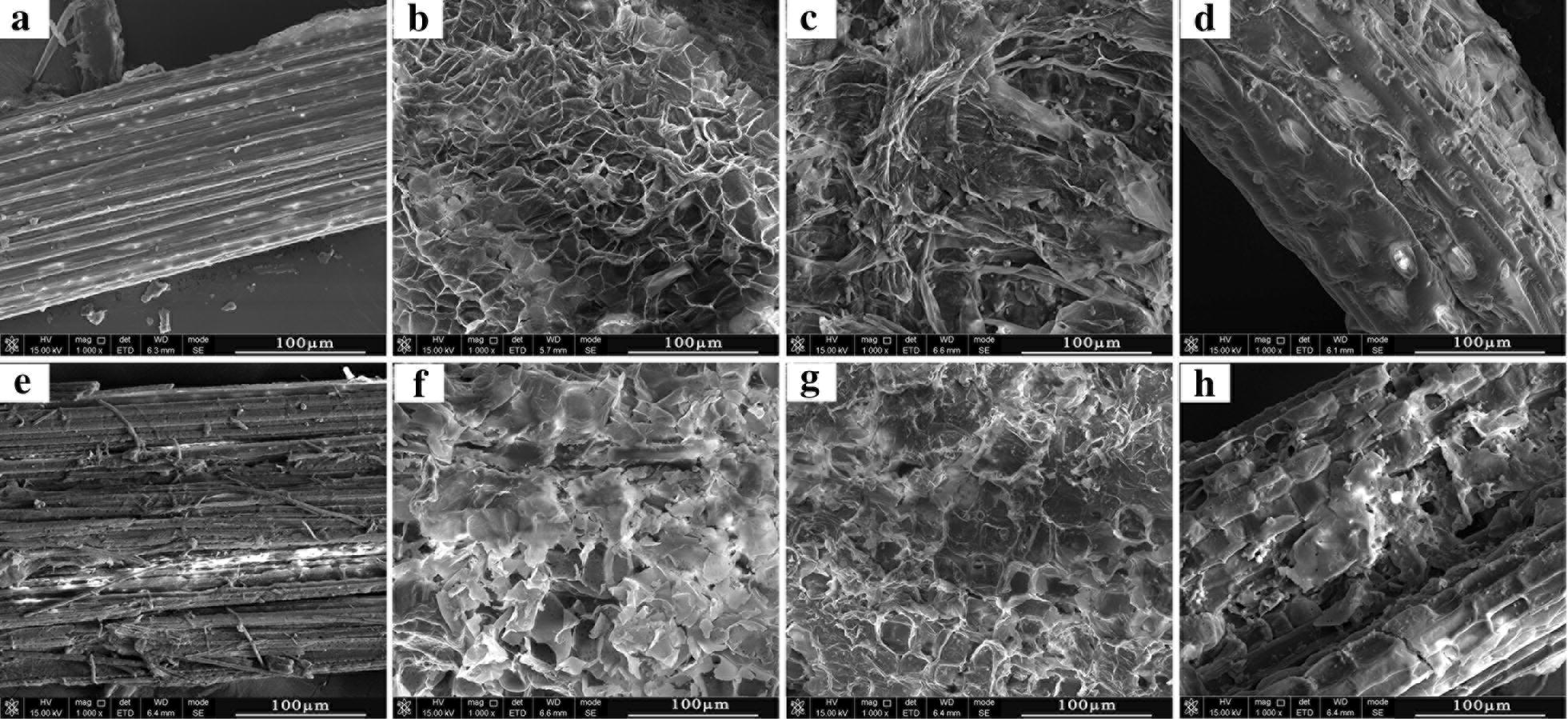
SEM images (×1000 magnification) of sweet sorghum straw. **a** Untreated. **b**–**d** NaOH, H_2_SO_4_ and H_2_O_2_ pretreated, respectively. **e** Untreated and enzyme hydrolyzed. **f**–**h** NaOH, H_2_SO_4_ and H_2_O_2_ pretreated and enzyme hydrolyzed, respectively. Scale bars: 100 µm

The surface morphology changes of SSS after pretreatment and enzymatic hydrolysis were investigated by using SEM. The untreated SSS showed a compact and rigid surface structure, and the only enzyme-hydrolyzed samples exhibited slight fiber splitting and surface abrasion (Fig. 3), which were consistent with our previous report described by Dong et al. [19]. The SEM image of NaOH-pretreated SSS exhibited more loose porous structures and specific surface areas that could effectively increase the accessibility of cellulase to biomass, which were strongly supported by the increased fluorescence intensity of dyed cellulase after alkali pretreatment in CLSM (Fig. 5). These morphological changes were caused by the reaction of alkali which leads to the removal of the surface lignin layer and release of cell shape of cellulose from the biomass matrix [19]. After hydrolysis of NaOH-pretreated SSS, much of the cell shape of cellulose was dissolved and more surface collapse was seen. The 2% H_2_SO_4_-pretreated samples showed a disorganized surface morphology characterized by the exposure of the loosely fibrous network. Ji et al. [48] investigated the structural changes in plant cell walls subjected to dilute acid pretreatment and found a sequence of pretreatment-induced deconstruction, including loss in the matrix between neighboring cell walls, removal of hemicelluloses and increased exposure of cellulose, thereby enhancing enzyme access to cellulose and further sugar yield. The image of hydrolyzed SSS after H_2_SO_4_ pretreatment showed slight surface collapse and hydrolysis of fibrous network, and the remaining structures could be lignin, minerals and other solid residues [49]. The H_2_O_2_ pretreatment caused the removal of surface wax and partial hemicellulose–lignin matrix, thus exhibiting a few internal fiber structures and some persistent solid residues still present after enzymatic hydrolysis.

### Visualization of enzymatic hydrolysis process of alkali-pretreated sweet sorghum straw

Based on the delignification capability and hydrolysis efficiency for SSS, the alkali-pretreated SSS was used for further enzymatic hydrolysis process study.

#### Real-time imaging on the enzymatic hydrolysis process of alkali-pretreated SSS

**Fig. 4:**
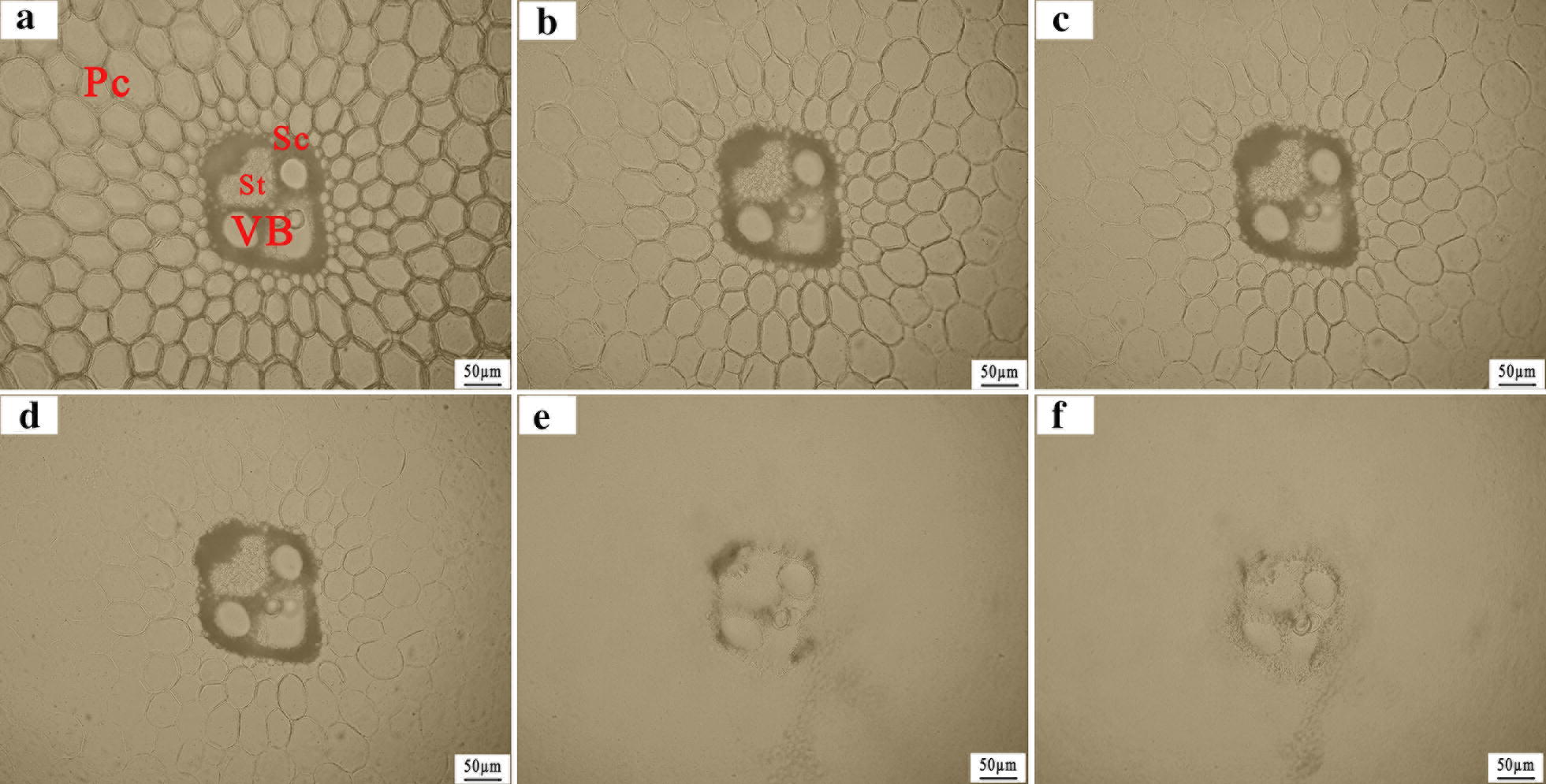
Alkali-pretreated sweet sorghum straw (transverse section, 20 µm in thickness) imaged in real time during enzymatic hydrolysis at room temperature (enzyme loading: 9 IU/g substrate). Image **a**–**f** were recorded with time intervals of 60 min using bright-field light microscopy. *Pc* parenchyma cells, *VB* vascular bundles, *Sc* sclerenchyma cells, *St* sieve tube. Scale bars: 50 µm

**Fig. 5:**
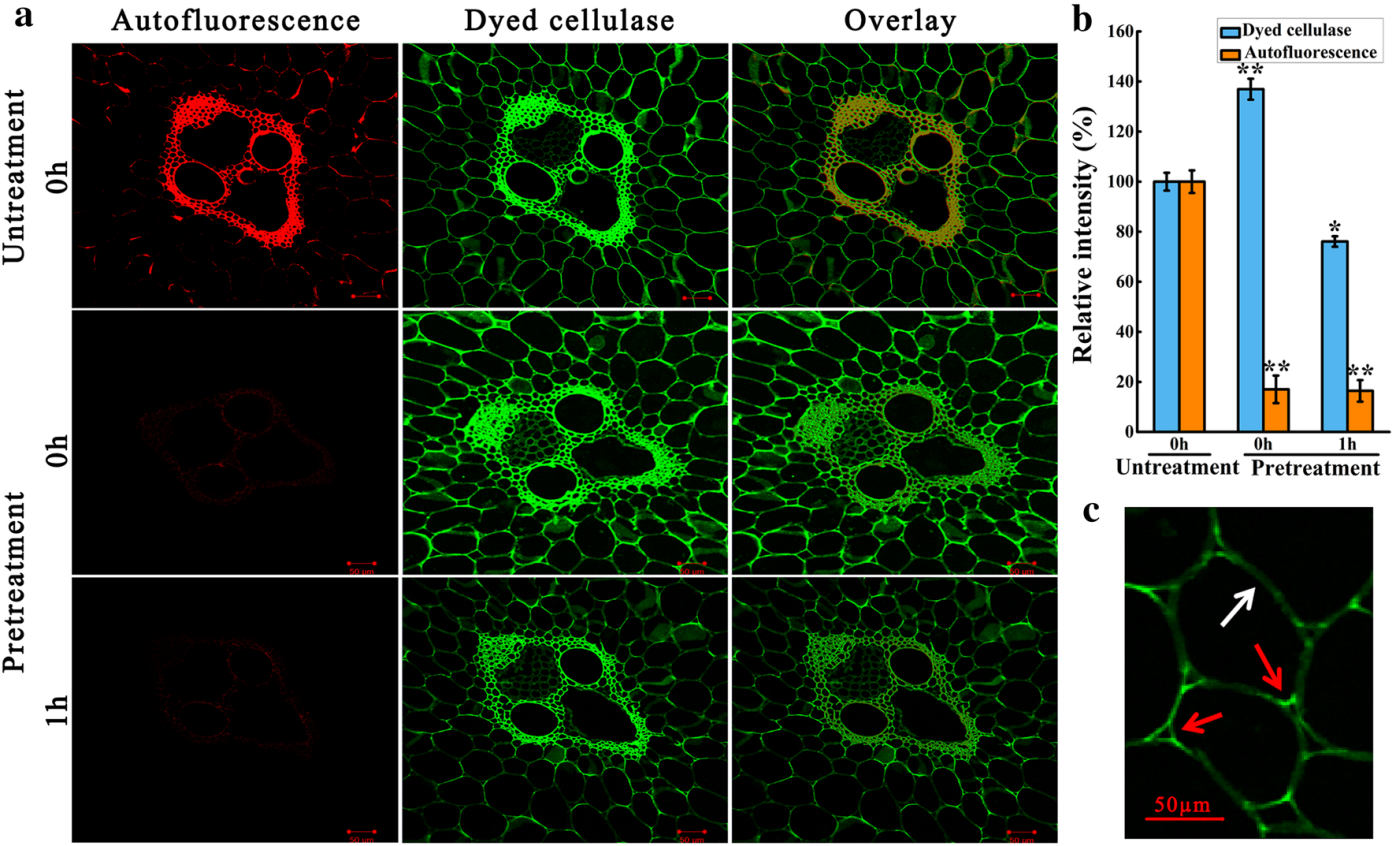
CLSM images of transverse section (10 µm in thickness). **a** The autofluorescence and dye-labeled fluorescence of cell walls (vascular bundle area) with or without alkali pretreatment. **b** The relative fluorescence intensities of dye-labeled cellulase and autofluorescence of samples at the same conditions, which were expressed as percentages compared with the intensities of fluorescence in untreated samples that were designated as 100%, respectively. **c** The changes in intensity of dye-labeled fluorescence between the corner (red arrows) and middle (white arrows) of alkali-pretreated parenchyma cell wall after hydrolysis of 1 h. Scale bars: 50 µm

Analyzing the enzymatic hydrolysis process of alkali**-**pretreated SSS allows us to visualize the spatial variation of hydrolysis rate and deepen the understanding of the detailed digestion manner of cellulase to cell walls. Therefore, we chose to focus on the vascular bundle areas, as this section contains a number of cell types. The transverse section of sweet sorghum stem showed the typical tissue structure of monocotyledons, including sclerenchyma cells (Sc) surrounding the vascular bundle (VB), parenchyma cells (Pc) and sieve tubes (St, Fig. 4a). Apparently, the hydrolysis rate in Pc far from VB was much faster than in Sc and VB, and the VB were degraded lastly (Additional file 1: Movie S1), indicating that the overall digestibility was negatively correlated with the residual lignin contents in pretreated cell walls. After 5 h of hydrolysis, the complete degradation of Pc was observed with the 9 IU/g substrate enzyme loading. In addition, we recorded the real-time digestion process in which the cellulase dissolved the cell walls in the same manner (Fig. 4 and Additional file 1: Movie S1). The enzymatic digestion firstly occurred from the middle of the cell walls and then toward the cell wall corners, which was also supported by the CLSM results (Fig. 5c). To the best of our knowledge, this study was the first report of the real-time imaging analysis of the enzymatic hydrolysis process in alkali-pretreated SSS.

#### Visualization of accessibility and digestibility of cellulase to alkali-pretreated cell walls during enzymatic hydrolysis

Although alkali pretreatment has been proved to be one of the most effective methods for biomass pretreatment, a detailed enzymatic hydrolysis process of cellulase to alkali-pretreated biomass is still lacking. Therefore, the CLSM was used to visualize the spatial changes in the accessibility and digestibility of cellulase to pretreated biomass, and the changes in content of heterogeneous components during hydrolysis were also visualized.

The detailed distribution of lignin autofluorescence in untreated SSS are presented in Fig. 5a. The Sc, VB and corners of Pc in natural SSS were highly lignified, thereby exhibiting strong autofluorescence. Ji et al. [48] reported that the raw material of *Miscanthus *× *giganteus* had a heterogeneous distribution of lignin component, clearly exhibiting high intensity in the metaxylem vessel, followed by Sc and Pc. Similarly, Hou et al. [50] also reported that the lignin components prefer accumulating in Sc, compound middle lamella and corners of Pc in rice straw. These observations were in agreement with the general plant anatomy. However, the alkali pretreatment significantly decreased the autofluorescence intensity of SSS (*P* < 0.01, Fig. 5a, b), indicating the occurrence of strong delignification. The extensive delignification in alkali-pretreated SSS was well supported by the disappearance of the bands at 1735 cm^−1^ and 1510 cm^−1^ in the FTIR spectra (Fig. 1a). In addition, the increased CrI in alkali-pretreated SSS also supported the occurrence of delignification, because the lignin was amorphous in the native biomass (Fig. 2). In particular, most of the lignin located in the corners of Pc was removed thoroughly, consistent with the fast hydrolysis of Pc observed in our study (Fig. 4). However, the autofluorescence intensity did not change significantly after 1 h of hydrolysis, indicating that the residual lignin in cell walls could not be degraded by cellulase (Fig. 5b).

Although the presence of lignin component was considered to be a significant restricting factor in enzymatic hydrolysis of biomass [51], the mechanism of lignin affects enzyme digestibility by a physical barrier on the cellulose surface or the nonproductive binding to enzyme remains open to debate. Ding et al. [14] reported that the cellulase binds strongly to nonlignified primary walls and more weakly to highly lignified sclerenchyma-type secondary walls. They demonstrated that the accessibility of untreated biomass to enzyme binding exhibited negative correlation with its lignin content, which could hinder the binding of cellulase to biomass. However, as can be seen from the untreated samples (Fig. 5a), the dye-labeled fluorescence was non-selectively distributed in the transverse sections of parenchyma cells and highly lignified vascular bundles, indicating the occurrence of nonproductive binding of cellulase to lignin. Similarly, the nonproductive binding of cellulase to lignin has been reported by previous studies [48, 52]. Thus, we can conclude that the effect of lignin on enzyme digestibility was mainly attributed to the nonproductive bindings of cellulase to lignin, thereby increasing the recalcitrance of the biomass.

As anticipated, the fluorescence intensity of dyed cellulase in alkali-pretreated samples significantly increased at the initial stage of digestion (0 h), indicating that the alkali pretreatment increased the cellulase absorption ability on biomass. This increase of absorption ability in biomass to cellulase was mainly caused by the removal of lignin and partial hemicellulose, thereby increasing the porosity in biomass and providing more sites for cellulase binding to substrate [53], which were in good agreement with the structural characterization results of SEM in alkali-pretreated SSS (Fig. 3b). After 1 h of hydrolysis, the dye-labeled fluorescence intensity declined (Fig. 5b), which was attributed to the dissociation of dyed cellulase absorbed in cellulose due to the partial digestion of cellulose [54]. In addition, the fluorescence intensity of dyed cellulase in the middle of the cell walls was lower than in the cell wall corners (Fig. 5c), indicating that the middle of cell walls were firstly digested during the enzymatic hydrolysis, thereby causing the dissociation of cellulase from cellulose [15].

Therefore, based on the detailed digestion process of plant cell walls and the spatial variation of hydrolysis rate in various plant tissues, the future of research aimed at enhancing biomass hydrolysis efficiency will primarily focus on the genetic engineering design of plant cell structure (including the types of cells or ratios of primary and secondary cell walls) or plant tissues at the organization level. In future, for biorefineries to produce biofuels from biomass economically and effectively, these findings from pretreatment and enzymatic hydrolysis process could be integrated into the conversion processes, thereby enhancing the overall biomass processing efficiency.

## Conclusion

Greater understanding of pretreatment and enzymatic hydrolysis of biomass is the key to lignocellulose processing. The structural characterization by XRD, FTIR and SEM and enzymatic hydrolysis indicated that the alkali pretreatment was the most effective method for delignification and enzymatic digestion among the dilute acid, alkali and hydrogen peroxide pretreatment of SSS. The visualization of the enzymatic hydrolysis process revealed that the cellulase dissolved the cell walls in a same manner and the overall digestibility was negatively correlated with the lignin contents of SSS. In addition, the efficient pretreatment significantly changed the spatial accessibility and digestibility of cellulase to lignocellulose, thereby improving the hydrolysis efficiency.

## Supplementary information

**Additional file 1: Movie S1.** Bright-field light microscopy of hydrolysis of alkali-pretreated transverse section showing the vascular bundle area digestion by cellulase for 6 h at room temperature. 10× lens, *x*/*y* = 2980/2272 pixel. The movie was played back at 1000 times actual hydrolysis speed.

## Data Availability

All data generated or analyzed during this study are included in this published article and its Additional file.

## Abbreviations

SSS: sweet sorghum straw
FTIR: Fourier transform infrared
XRD: X-ray diffractogram
SEM: scanning electron microscopy
CLSM: confocal laser scanning microscopy
FPU: filter paper activity
FITC: fluorescein isothiocyanate
DMSO: dimethyl sulfoxide
CrI: crystallinity index
Pc: parenchyma cells
VB: vascular bundles
Sc: sclerenchyma cells
St: sieve tube
